# Longitudinal immune cell monitoring identified CD14^++^ CD16^+^ intermediate monocyte as a marker of relapse in patients with ANCA-associated vasculitis

**DOI:** 10.1186/s13075-020-02234-8

**Published:** 2020-06-16

**Authors:** Kotaro Matsumoto, Katsuya Suzuki, Keiko Yoshimoto, Noriyasu Seki, Hideto Tsujimoto, Kenji Chiba, Tsutomu Takeuchi

**Affiliations:** 1 Division of Rheumatology, Department of Internal Medicine, Keio University School of Medicine, 35 Shinanomachi, Shinjuku-ku, Tokyo, Japan; 2 Clinical and Translational Research Center, Keio University Hospital, 35 Shinanomachi, Shinjuku-ku, Tokyo, Japan; 3 Mitsubishi Tanabe Pharma Corporation, 1000, Kamoshida-cho, Aoba-ku, Yokohama, Kanagawa Japan

**Keywords:** Anti-neutrophil cytoplasmic antibody-associated vasculitis, Immuno-phenotyping, Intermediate monocyte, Plasma cell

## Abstract

**Background:**

Anti-neutrophil cytoplasmic antibody (ANCA)-associated vasculitis (AAV) is an autoimmune disease that affects small- to medium-sized blood vessels. Despite treatments having been improved, patients often experience disease relapses. It remains unclear how the immune cells involve in the development of vasculitis and how they fluctuate over the course of treatment. In this study, we aimed to identify the immune subsets and serum cytokines associated with disease relapse by comprehensive immuno-phenotyping in AAV patients.

**Methods:**

We reviewed consecutive patients (*n* = 29) from Keio University Hospital who had been newly diagnosed with AAV from January 2015 to February 2019 and chronologically followed until 52 weeks. Numbers of circulating T cells, B cells, monocytes, and granulocytes were analyzed by flow cytometry (FACS). Serum levels of cytokines were measured by electrochemiluminescence enzyme immunoassay. Clinical information was obtained from patients’ records and association with time-course changes in immuno-phenotypes and serum levels of cytokines were assessed.

**Results:**

Comprehensive immuno-phenotyping data from 161 samples from 29 AAV patients at diagnosis; at weeks 4, 12, 24, and 52 of treatment; and at time of major relapse were examined. FACS analysis from patients with relapse revealed that CD14^++^ CD16^+^ intermediate monocytes and plasma cells concomitantly changed associated with disease relapse, which were independent from treatment regimen, ANCA status, or disease phenotype. In particular, the number of CD14^++^ CD16^+^ intermediate monocytes at relapse was significantly higher than that in remission or in healthy controls. Serum cytokine measurement revealed that changes of monocyte-derived proinflammatory cytokines such as IL-1β, IL-6, IL-8, and TNF-α were associated with disease status.

**Conclusions:**

Chronological changes in CD14^++^ CD16^+^ intermediate monocyte counts can be a marker of disease relapse in AAV patients.

## Background

Anti-neutrophil cytoplasmic antibody (ANCA)-associated vasculitis (AAV) is an autoimmune disease that affects small- to medium-sized blood vessels and causes vascular inflammation and multiple organ damage [1, 2]. AAV is primarily managed with high-dose glucocorticoid in combination with cyclophosphamide, rituximab, or other conventional disease-modifying anti-rheumatic drugs (csDMARDs) [3–5]. Despite improvements following the introduction of cyclophosphamide- or rituximab-based treatment, however, patients often experience disease relapses [4–7].

Several lines of evidence from genetic and clinical research have revealed immune cells related to AAV pathogenesis. The results of genome-wide association studies suggest that human leukocyte antigen (HLA) genes including both major histocompatibility (MHC) class I and class II alleles are associated with disease susceptibility of AAV [8, 9]. Previous reports have shown abnormalities related to innate and adaptive immune cells are involved in the pathogenesis of AAV. For example, antigen-specific T and B cells are expanded, which produce ANCA [10–12]. ANCA subsequently leads neutrophils and monocytes to exposure of proteinase 3 (PR3) and myeloperoxidase (MPO) on their surface, allowing their recognition of ANCA [13]. Other reports have shown proliferation of circulating CD4^+^ T [14, 15], follicular helper T (Tfh) [16] and activated CD8^+^ T cells [17], and defects of regulatory T cells [18]. Previous reports showed that serum calprotectin and urinary soluble CD163 could be potential biomarkers, however, the exact role of monocytes in AAV has been unclear [19, 20]. Recently, we reported a comprehensive analysis using immuno-phenotyping to reveal that AAV patients have combined features of antibody production, cytotoxic activity, and neutrocytosis/lymphocytopenia [21]. Proportions of HLA-DR^+^ CD4^+^ T cells, HLA-DR^+^ CD8^+^ T cells, plasma cells, plasmablasts, CD14^++^ CD16^+^ intermediate monocytes, eosinophils, and neutrophils were increased in AAV compared to healthy controls (HCs) [21]. Various studies exist; however, identifying the specific cell subsets that drive the disease relapse has been challenging.

Here, therefore, we aimed to identify the immune subsets and serum cytokines associated with disease relapse by comprehensive immuno-phenotyping in AAV patients.

## Patients and methods

### Patients and healthy controls

Consecutive patients with newly diagnosed AAV (*n* = 29) who visited Keio University Hospital and fulfilled the 2012 Revised International Chapel Hill Consensus Conference Nomenclature [22] for granulomatosis with polyangiitis (GPA) and microscopic polyangiitis (MPA) between January 2015 and February 2019 and HCs (*n* = 18) were enrolled.

All patients received glucocorticoid therapy at an initial dose equivalent to 0.6–1.0 mg prednisolone (PSL) per kilogram per day, which was tapered based on previously reported clinical trials [4, 5]. As induction therapy, cyclophosphamide, rituximab, or others were selected by the attending physician in routine practice. Intravenous cyclophosphamide pulse was given at 10–15 mg/kg for 2 to 4 weeks in 6 cycles, and rituximab was given at 375 mg/m^2^ body surface area per week for 4 cycles [3]. Patients who received rituximab as induction therapy were usually treated with rituximab as maintenance therapy. Patients who received cyclophosphamide were treated with azathioprine, methotrexate, or tacrolimus as maintenance therapy.

We confirmed that HCs did not have any autoimmune disease, severe allergic disorder, malignancy, or infection.

This study was approved by the research ethics committee of Keio University School of Medicine (#20140335) and was conducted according to the Declaration of Helsinki. Informed consent was obtained from all participants.

### Clinical assessment

Clinical information was obtained from the patients’ records, including organ involvement of the ear, nose, and throat (ENT); central nervous system (CNS); peripheral nervous system (PNS); and kidney and interstitial lung disease (ILD), and data from laboratory studies of erythrocyte sedimentation rate (ESR), white blood cell count, hemoglobin, platelet count, ANCA titer (by chemiluminescence enzyme immunoassay; CLEIA), rheumatoid factor (RF), IgG, and C-reactive protein (CRP) in serum. Patients were followed until 52 weeks of treatment, until they died or were withdrawn, and information on disease relapse was examined.

We evaluated the total Birmingham Vasculitis Activity Score (BVAS) and the components of BVAS for each organ involvement. Remission was defined as an absence of clinical disease activity, as indicated by a BVAS of 0 that was maintained for at least 2 months [23]. Relapse was defined as the recurrence or new appearance of any disease activity, as reflected by the BVAS following disease remission requiring reinduction therapy [4]. Relapse of ILD was defined as the reappearance of new bilateral ground-glass opacities on high-resolution computed tomography requiring treatment intensification [24]. Severe infectious diseases were designated grade ≥ 3 based on the Common Terminology Criteria for Adverse Events V.4.0 [25].

### Flow cytometry (FACS) analysis for immuno-phenotyping

We collected heparinized peripheral blood samples from patients with AAV at diagnosis; at weeks 4, 12, 24, and 52 of treatment; and at time of major relapse. Because corticosteroids alter the number of peripheral immune cells in peripheral blood [26], immuno-phenotyping was carried out prior to receipt of high-dose corticosteroids at baseline and relapse. FACS analysis was performed according to MIFlowCyt [27] and was conducted on LSRFortessa™ X-20 (BD Biosciences, NJ, USA) without delay after collecting samples. One hundred microliters of heparinized blood samples were stained with antibodies (Supplementary Fig. 1); then, the staining samples were fixed by (PhosflowTM Lyse/Fix buffer, BD, NJ, USA). We used Flow-count Fluorospheres (BECKMAN COULTER, CA, USA) to acquire standardized number of immune cells. Data were analyzed by 3 individual authors blinded to the sample details using FlowJo v.10.1 Software (Tree Star, Stanford University, CA, USA).

The phenotypes of immune cell subsets were defined based on the Human Immunology Project protocol [28]. We modified the definition of plasmablast and plasma cell based on other references [29, 30], CD19^+^ CD27^+^ IgD^−^ CD20^−^ CD38^+^ CD138^+^ as plasmablast and CD19^+^ CD27^+^ IgD^−^ CD20^−^ CD38^+^ CD138^++^ as plasma cell. When we performed FACS analysis, micro-sized cells were gated out, instead of using a dead/live marker. Details of the gating strategy are shown in Supplementary Fig. 1.

### Cytokine measurement

Levels of interferon (IFN)-γ, interleukin (IL)-1β, IL-6, IL-8, IL-10, and tumor necrosis factor (TNF)-α in serum were determined using a commercial electrochemiluminescence assay (Meso Scale Discovery, MD, USA) according to the manufacturer’s protocol. Serum samples were stored at − 80 °C prior to assay.

### Statistical analysis

All analyses were conducted using SPSS Statistics version 26.0 (IBM Corp., NY, USA), JMP version 14.0 (SAS Institute, NC, USA) or GraphPad Prism software version 8.0 (GraphPad, CA, USA). Continuous data are expressed as median (IQR), and categorical data as number and/or percentage. Descriptive statistics were used to summarize the data. Continuous variables were compared using the Mann-Whitney *U* test, and categorical variables using the chi-squared test. Longitudinal data was analyzed using generalized estimating equations (GEE) [31–33], Wilcoxon signed rank sum test, repeated measures analysis of variance (ANOVA), and post hoc test. For GEE, the number of peripheral immune cells were normalized using *z*-score. Then, univariate GEE association over time for normalized number of peripheral immune cells and levels of serum cytokines with BVAS was analyzed. *P* values less than 0.05 were considered significant.

## Results

### Baseline characteristics, treatment, and therapeutic prognosis of patients

We collected FACS data and clinical profiles from 29 AAV patients who were followed longitudinally across a total of 161 visits. All patients were treated with glucocorticoid-based treatment. Ten patients (34%) were treated with cyclophosphamide and 8 (28%) were treated with rituximab. The other 11 patients (38%) were treated with PSL as monotherapy or in combination with csDMARDs (azathioprine, 9; methotrexate, 1). All patients received rituximab as maintenance therapy when they reached remission by using rituximab as induction therapy. When patients reached remission with cyclophosphamide induction, they received azathioprine, methotrexate, or tacrolimus as maintenance therapy. A small number of patients were unable to use immunosuppressive drugs as maintenance therapy due to severe infection or other adverse events.

Baseline characteristics, treatment, and therapeutic prognosis of the patients treated with each regimen and HCs are summarized in Tables 1 and 2. Patients treated without using cyclophosphamide or rituximab tended to be older (cyclophosphamide vs rituximab vs others: 68 [52–75] vs 61 [50–76] vs 79 [64–83] years), higher proportion of MPA (50 vs 50 vs 64%), and lower BVAS (12 [12–17] vs 9 [9–16] vs 10 [5–14]). Of the 29 patients, 9 patients (26%) experienced relapse during the 52-week follow-up period.

**Table 1:** Baseline characteristics, treatment, and effects of treatment in AAV patients

Variable	AAV, *n* = 29	Induction treatment			HC, *n* = 18
		IVCY, *n* = 10	RTX, *n* = 8	Others, *n* = 11	
Baseline demographic					
Age at diagnosis, years	70 (57–79)	68 (52–75)	61 (50–76)	79 (64–83)	51 (46–60)
Male, *n* (%)	13 (45)	6 (60)	2 (25)	5 (45)	7 (39)
PR3-ANCA^+^ GPA, *n* (%)	7 (24)	3 (30)	2 (25)	2 (18)	
MPO-ANCA^+^ GPA, *n* (%)	5 (17)	2 (20)	1 (13)	2 (18)	
ANCA^−^ GPA, *n* (%)	1 (3)	0 (0)	1 (13)	0 (0)	
MPO-ANCA^+^ MPA, *n* (%)	16 (55)	5 (50)	4 (50)	7 (64)	
BVAS	12 (8.5–15)	12 (12–17)	9 (9–16)	10 (5–14)	
Organ involvement					
Ear, nose, throat, *n* (%)	13 (45)	5 (50)	3 (38)	5 (45)	
CNS, *n* (%)	7 (24)	2 (20)	3 (38)	2 (18)	
PNS, *n* (%)	5 (17)	1 (10)	2 (25)	2 (18)	
Kidney, *n* (%)	12 (41)	5 (50)	3 (38)	4 (36)	
ILD, *n* (%)	17 (59)	5 (50)	4 (50)	8 (73)	
Laboratory tests					
ESR, mm/h	102 (47–124)	105 (93–123)	53 (17–119)	121 (61–128)	
WBC, × 10^3^ cells/μL	9.2 (7.0–14)	14 (8.7–15)	8.3 (5.4–9.0)	9.2 (6.1–15)	
Hemoglobin, g/dL	11 (10–13)	11 (8.7–12)	13 (9.9–15)	11 (10–12)	
Platelet, × 10^4^ cells/μL	33 (25–41)	38 (28–41)	29 (20–35)	32 (26–49)	
CRP, mg/dL	5.5 (1.2–9.4)	8.5 (3.8–13)	1.1 (0.3–7.7)	5.5 (4.4–8.3)	
IgG, g/dL	1.8 (1.3–2.0)	1.8 (1.2–2.0)	1.6 (0.9–2.1)	1.8 (1.5–2.2)	
RF, IU/L	56 (21–146)	43 (8.8–146)	33 (23–143)	65 (37–301)	
MPO/PR3-ANCA, IU/L	48 (17–189)	44 (17–300)	40 (9.5–82)	77 (25–221)	
PSL dose					
Initial PSL dose, mg/day	45 (33–60)	59 (45–63)	43 (30–59)	40 (30–50)	
PSL dose at week 24, mg/day	11 (10–16)	14 (10–33)	11 (7.6–12)	13 (10–16)	
PSL dose at week 52, mg/day	8 (6–11)	9 (5.5–16)	8 (4.5–9.3)	8 (6–14)	
Maintenance therapy	AZA, 12 (41); RTX, 6 (21); MTX, 2 (7); Tac, 2 (7)	AZA. 5 (50); Tac, 2 (20)	RTX, 6 (75)	AZA, 7 (64); MTX, 2 (18)	
Outcome					
Relapse, *n* (%)	10 (34)	3 (30)	3 (38)	3 (27)	
Severe infection, *n* (%)	10 (34)	5 (50)	2 (25)	3 (27)	
Death, *n* (%)	2 (7)	1 (10)	0 (0)	1 (9)	

Continuous data are expressed as median (IQR), and categorical data as number and/or percentage. *AAV* ANCA-associated vasculitis, *GPA* granulomatosis with polyangiitis, *MPA* microscopic polyangiitis, *WBC* white blood cell, *CRP* C-reactive protein, *RF* rheumatoid factor, *PSL* prednisolone, *IVCY* intravenous cyclophosphamide, *RTX* rituximab, *AZA* azathioprine, *MTX* methotrexate, *Tac* tacrolimus

**Table 2:** Baseline characteristics, treatment, and effects of treatment in AAV patients with responder and non-responder

Variable	Without relapse, *n* = 20	With relapse, *n* = 9	*P* value
Baseline demographic			
Age at diagnosis, years	67 (55–78)	75 (65–80)	0.27
Male, *n* (%)	11 (58)	2 (22)	0.070
PR3-ANCA^+^ GPA, *n* (%)	4 (20)	3 (33)	0.65
MPO-ANCA^+^ GPA, *n* (%)	3 (15)	2 (22)	0.64
ANCA^−^ GPA, *n* (%)	1 (5)	0 (0)	0.38
MPO-ANCA^+^ MPA, *n* (%)	12 (60)	4 (44)	0.44
BVAS	11 (8.3–16)	12 (8.5–16)	0.74
Organ involvement			
Ear, nose, throat, *n* (%)	7 (35)	6 (67)	0.11
CNS, *n* (%)	4 (20)	3 (33)	0.43
PNS, *n* (%)	2 (10)	3 (33)	0.13
Kidney, *n* (%)	10 (50)	2 (22)	0.15
ILD, *n* (%)	11 (55)	5 (56)	0.98
Laboratory tests			
ESR, mm/h	102 (48–125)	106 (43–127)	0.77
WBC, × 10^3^ cells/μL	9.9 (7.1–14)	9.0 (6.3–12)	0.46
Hemoglobin, g/dL	11 (9.8–13)	11 (9.4–13)	0.51
Platelet, × 10^4^ cells/μL	33 (26–42)	35 (19–40)	0.51
CRP, mg/dL	5.2 (1.6–8.5)	8.3 (0.8–11)	0.57
IgG, mg/dL	1.8 (1.3–2.0)	1.8 (1.2–2.3)	0.55
RF, IU/L	56 (26–188)	37 (15–93)	0.28
MPO/PR3-ANCA, IU/L	79 (19–276)	40 (14–51)	0.14
PSL dose			
Initial PSL dose, mg/day	50 (40–60)	35 (30–58)	0.28
PSL dose at week 24, mg/day	10 (9–13)	17 (11–35)	**0.026**
PSL dose at week 52, mg/day	7 (5–9)	10 (10–16)	**0.0071**
Immunosuppressive drugs			
Induction therapy	IVCY, 7 (35); RTX, 5 (25); AZA, 8 (40)	IVCY, 3 (33); RTX, 3 (33); AZA, 1 (11); MTX, 1 (11)	–
Maintenance therapy until relapse	RTX, 5 (25); AZA, 10 (50); MTX, 1 (5); Tac, 2 (10)	RTX, 1 (11); AZA, 2 (22); MTX, 1 (11)	–
Reinduction therapy	–	IVCY, 3 (33); RTX, 2 (22)	–
Maintenance therapy after reinduction therapy	–	RTX, 1 (11); AZA, 2 (22); Tac, 1 (11)	–
Outcome			
Severe infection, *n* (%)	4 (20)	6 (67)	**0.015**
Death, *n* (%)	1 (5)	1 (11)	0.56

Continuous data are expressed as median (IQR), and categorical data as number and/or percentage. Continuous variables were compared using the Mann-Whitney *U* test, and categorical variables using the chi-squared test. Values in bold are statistically significant (*P* <  0.05). *AAV* ANCA-associated vasculitis, *GPA* granulomatosis with polyangiitis, *MPA* microscopic polyangiitis, *CNS* central nervous system, *PNS* peripheral nervous system, *ESR* erythrocyte sedimentation rate, *WBC* white blood cell, *CRP* C-reactive protein, *RF* rheumatoid factor, *PSL* prednisolone, *IVCY* intravenous cyclophosphamide, *RTX* rituximab, *AZA* azathioprine, *MTX* methotrexate, *Tac* tacrolimus

### Changes in immuno-phenotyping associated with disease relapse

To assess changes in the numbers of peripheral immune cells at remission and at relapse phase, we longitudinally profiled the immuno-phenotyping data of 9 patients who achieved remission then relapsed. We followed the immuno-phenotyping data of these patients until 52 weeks of treatment and calculated the correlation score between the fluctuation in each immune cell and change in disease activity.

We next examined the longitudinal associations between normalized number of peripheral immune cells and BVAS (Table 3). Univariate GEE analysis showed that changes in BVAS were significantly associated with changes in CD14^++^ CD16^+^ intermediate monocytes (*β* = 0.82, 95%CI 0.25–1.4, *P* = 0.005), CD14^++^ CD16^−^ classical monocytes (*β* = 0.61, 95%CI 0.018–1.2, *P* = 0.043), and eosinophils (*β* = 0.56, 95%CI 0.026–1.1, *P* = 0.040). Additionally, plasma cells (*β* = 0.54, 95%CI: − 0.037 to 1.1, *P* = 0.067) tended to be associated with total BVAS, while that of other cell subsets did not show any notable correlation.

**Table 3:** Univariate GEE, longitudinal associations of the number of peripheral immune cells, and levels of serum cytokines with BVAS

	BVAS		
	*β*	95%CI	*P* value
CD4+ T	0.11	− 0.27 to 0.48	0.58
Th1	0.072	− 0.38 to 0.52	0.76
Th1 HLA-DR+	0.090	− 0.52 to 0.69	0.77
Th2	0.092	− 0.34 to 0.52	0.67
Th2 HLA-DR+	− 0.074	− 0.75 to 0.61	0.83
Th17	0.098	− 0.15 to 0.35	0.45
Th17 HLA-DR+	0.29	− 0.060 to 0.65	0.10
Treg	− 0.041	− 0.30 to 0.22	0.76
Treg HLA-DR+	0.030	− 0.35 to 0.41	0.88
Tfh	0.12	− 0.15 to 0.40	0.38
CD8+ T	0.23	− 0.15 to 0.62	0.24
CD8 HLA-DR+	0.24	− 0.18 to 0.66	0.27
CD19+ B	0.32	− 0.041 to 0.69	0.082
Plasma cell	0.54	− 0.037 to 1.1	0.067
Monocyte	0.66	0.048–1.3	**0.035**
CD14++ CD16-	0.61	0.018–1.2	**0.043**
CD14++ CD16+	0.82	0.25–1.4	**0.005**
CD14+ CD16+	0.62	− 0.21 to 1.5	0.14
Eosinophil	0.56	0.026–1.1	**0.040**
Neutrophil	− 0.066	− 0.57 to 0.44	0.80
IFN-γ	0.51	− 0.13 to 1.1	0.12
IL-1β	1.0	0.57–1.5	**< 0.001**
IL-6	0.89	0.56–1.2	**< 0.001**
IL-8	0.60	0.10–1.1	**0.018**
IL-10	0.51	− 0.077 to 1.1	0.088
TNF-α	0.91	0.43–1.4	**< 0.001**

Longitudinal relationship of the number of peripheral immune cells and levels of serum cytokines with BVAS. Values in bold are statistically significant (*P* < 0.05). *Th* helper T, *Treg* regulatory T, *Tfh* follicular helper T, *IFN* interferon, *IL* interleukin

**Fig. 1:**
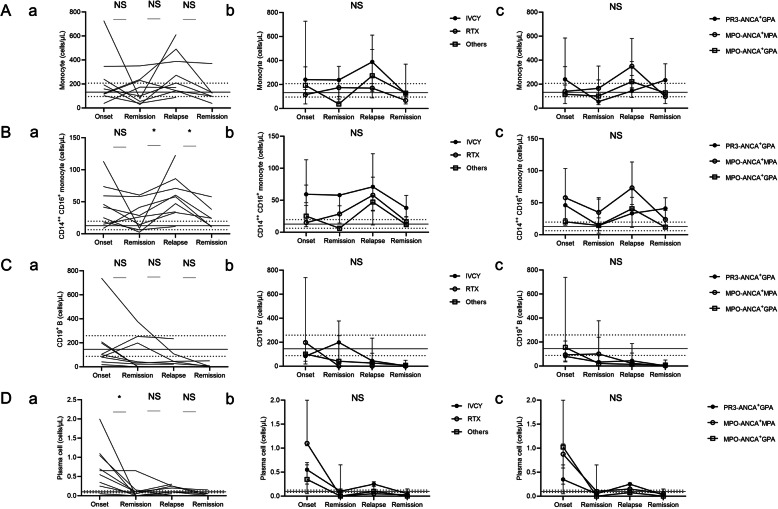
Correlation analysis between disease activity and immune cell numbers in AAV patients with relapse. Changes in **A** monocytes, **B** CD14^++^ CD16^+^ intermediate monocytes, **C** CD19^+^ B cells, and **D** plasma cells in AAV patients with relapse (*n* = 9). Duration from onset to 1st remission, onset to relapse, and onset to 2nd remission were 7.4 (4.4–12), 15 (12–37), and 24 (19–43) weeks. **A**–**D** Lines showed the median (IQR) number of immune cell subsets in healthy controls. **A**-a, **B**-a, **C**-a, **D**-a Individual data were shown. **A**, **B** *P <  0.05 for analysis using Wilcoxon signed rank sum test. The difference among treatment regimens (**A**-b, **B**-b, **C**-b, and **D**-b) and disease phenotypes (**A**-c, **B**-c, **C**-c, and **D**-c) were shown by the median and IQR and analyzed by repeated measures ANOVA and post hoc Friedman test

Then, we compared absolute number of circulating immune cells among onset, 1st remission, relapse, and 2nd remission using Wilcoxon signed rank sum test. As shown in Table 4, we found that the number of plasma cell and eosinophil at onset was higher than that at remission. CD14^++^ CD16^+^ intermediate monocytes at relapse were higher than that at remission. The number of CD14^++^ CD16^+^ intermediate monocytes at relapse was 3.2 times greater than that in HCs (Fig. 1A-a). The number of plasma cells at relapse was comparable to those in remitted patients and HCs (Fig. 1B-a).

**Table 4:** Comparison of longitudinal peripheral immune cells in AAV patients with relapse

Immune cell subtype (cells per μL)	HC	Onset	1st Rem	Relapse	2nd Rem	*P* value		
	*n* = 18	*n* = 9	*n* = 9	*n* = 9	*n* = 6	Onset vs 1st Rem	1st Rem vs relapse	Relapse vs 2nd Rem
CD19+ B	162 (104–242)	101 (60–203)	33 (1.8–226)	27 (0.4–76)	1.6 (0.2–18)	0.30	0.46	0.57
Plasma cell	0.1 (0.1–0.2)	0.7 (0.3–1.1)	0.05 (0–0.13)	0.1 (0.08–0.3)	0.03 (0–0.08)	**0.036**	0.95	0.86
Monocyte	194 (130–250)	159 (109–293)	100 (46–234)	208 (144–440)	110 (82–188)	0.29	0.087	0.12
CD14++ CD16-	152 (99–187)	107 (56–225)	65 (32–166)	134 (89–346)	72 (53–153)	0.34	0.12	0.14
CD14++ CD16+	17 (10–23)	42 (17–67)	26 (7.3–50)	58 (34–79)	24 (11–43)	0.20	**0.027**	**0.049**
CD14+ CD16+	8.1 (4.7–17)	5.8 (2.8–13)	2.9 (2.2–6.7)	7.4 (4.4–10)	5.0 (3.5–9.8)	0.095	0.13	0.44

Wilcoxon signed rank sum test was used to compare the point of onset, 1st remission, relapse, and 2nd remission. *Th* helper T, *Treg* regulatory T, *Tfh* follicular helper T

The difference among treatment regimens (Fig. 1A-b, B-b, C-b, and D-b) and disease phenotypes (Fig. 1A-c, B-c, C-c, and D-c) was analyzed by repeated measures ANOVA and post hoc Friedman test. There was no significant difference in changes of monocytes (Fig. 1A-b), CD14^++^ CD16^+^ intermediate monocytes (Fig. 1B-b), CD19^+^ B cells (Fig. 1C-b), and plasma cells (Fig. 1D-b) among treatment regimens. In addition, the levels of monocytes (Fig. 1A-c), CD14^++^ CD16^+^ intermediate monocytes (Fig. 1B-c), CD19^+^ B cells (Fig. 1C-c), and plasma cells (Fig. 1D-c) were also independent on ANCA serotype or disease phenotype.

We found that only 3 (33%) patients (cases 1, 2, 9) showed a return of MPO/PR3-ANCA at relapse, while the remaining 6 patients remained ANCA-negative at relapse (Table 5). Of the 3 patients with ANCA return at relapse, all were GPA with CNS involvement, and 2 (67%) were PR3-ANCA-positive.

**Table 5:** Summary of 9 AAV patients with relapse

Case no.	Age (years)/sex	Induction Tx	At onset		At remission	At relapse	
			ANCA/disease	Organ involvement	ANCA status	Organ involvement	ANCA status
Case 1	38/M	PSL+IVCY	PR3^+^ GPA	CNS, ENT	Negative	CNS, ENT	Positive
Case 2	57/F		PR3^+^ GPA	CNS, ENT	Negative	CNS, ENT	Positive
Case 3	74/M		MPO^+^ MPA	ILD, kidney	Negative	ILD, kidney	Negative
Case 4	76/F	PSL+RTX	MPO^+^ MPA	ILD, kidney, PNS	Negative	ILD, kidney, PNS	Negative
Case 5	78/F		MPO^+^ GPA	ENT, ILD, PNS	Negative	ILD, PNS	Negative
Case 6	75/F		MPO^+^ MPA	PNS	Negative	PNS	Negative
Case 7	90/M	PSL+AZA	MPO^+^ MPA	ILD, kidney	Negative	Kidney	Negative
Case 8	82/F	PSL monotherapy	PR3^+^ GPA	ENT, ILD	Negative	ILD	Negative
Case 9	72/F	PSL monotherapy	MPO^+^ GPA	CNS, ENT	Negative	CNS, ENT	Positive

*PSL* prednisolone, *IVCY* intravenous cyclophosphamide, *RTX* rituximab, *AZA* azathioprine, *MPA* microscopic polyangiitis, *GPA* granulomatosis with polyangiitis, *ENT* ear, nose, throat, *PNS* peripheral nervous system, *ILD* interstitial lung disease

### Chronological changes in immuno-phenotyping in AAV patients without relapse

To assess the difference between patients with and without relapse on CD14^++^ CD16^+^ intermediate monocytes and plasma cells, cell numbers in 20 patients successfully treated without relapse were followed at diagnosis and at weeks 4, 12, 24, and 52 of treatment (Supplementary Fig. 2). Changes in PSL dose and BVAS were shown (Supplementary Fig. 2A and B). The number of CD14^++^ CD16^+^ intermediate monocytes in patients remained high compared with HCs after 52 weeks of treatment (Supplementary Fig. 2C). In contrast, the number of plasma cells decreased to normal levels as well as in HCs after treatment (Supplementary Fig. 2D).

### Chronological changes in humoral factors in AAV patients with relapse

Given the significant correlation between the number of CD14^++^ CD16^+^ intermediate monocytes and disease activity, we evaluated serum levels of the proinflammatory cytokines associated with monocyte activation, namely IFN-γ, IL-1β, IL-6, IL-8, IL-10, and TNF-α. Results of univariate GEE analysis for longitudinal associations between serum cytokine levels and BVAS showed significant associations for BVAS and IL-1β (*β* = 1.0, 95%CI 0.57–1.5, *P* <  0.001), IL-6 (*β* = 0.89, 95%CI 0.56–1.2, *P* <  0.001), IL-8 (*β* = 0.60, 95%CI 0.10–1.1, *P* = 0.018), and TNF-α (*β* = 0.91, 95%CI 0.43–1.4, *P* <  0.001) (Table 3).

**Fig. 2:**
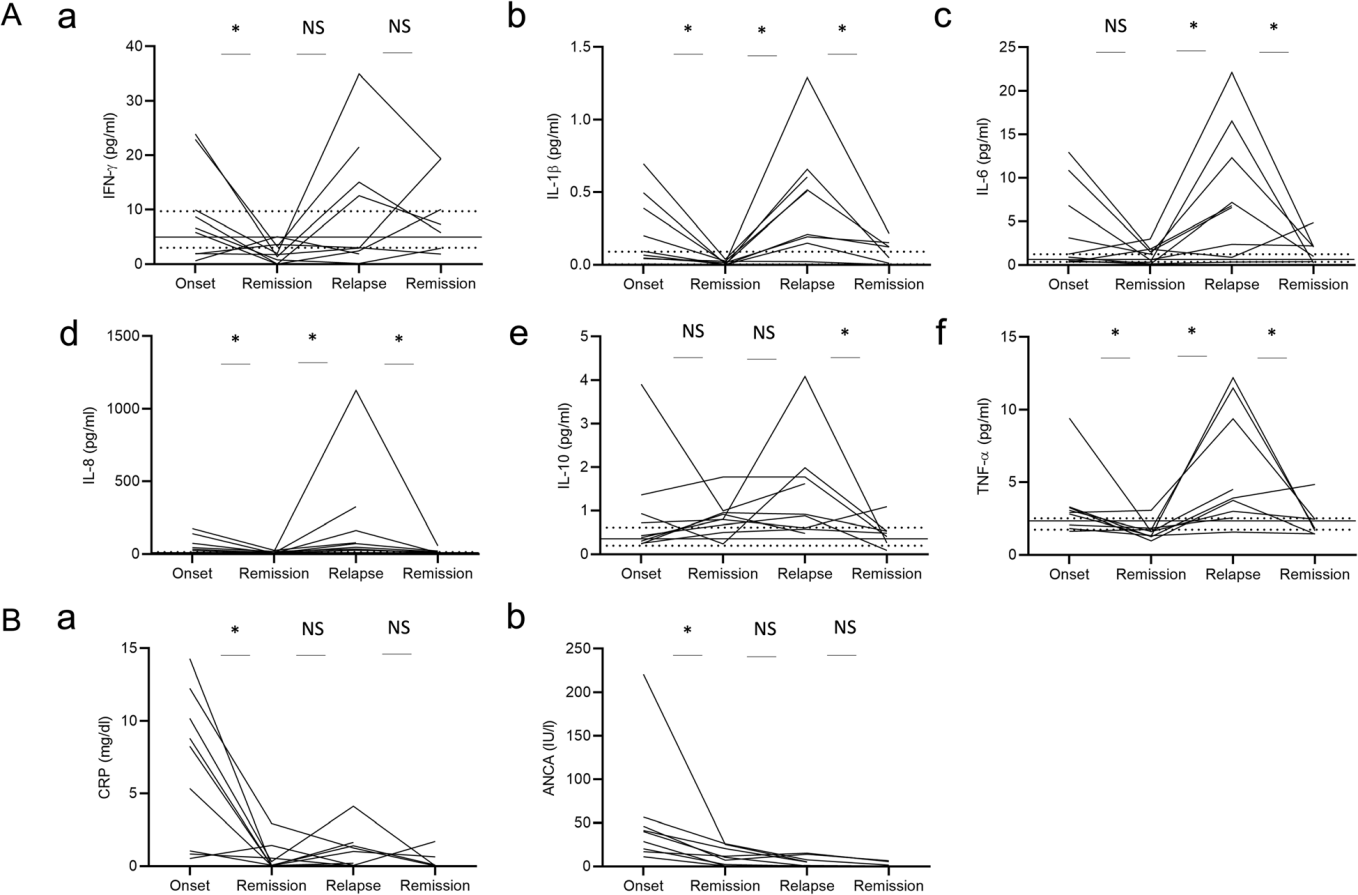
Changes in humoral factors in AAV patients with relapse. **A** Serum cytokines of (a) IFN-γ, (b) IL-1β, (c) IL-6, (d) IL-8, (e) IL-10, and (f) TNF-α, and **B** laboratory findings of (a) CRP and (b) ANCA titer in AAV patients with disease relapse (*n* = 9). **A** Lines showed the median (IQR) number of immune cell subsets in healthy controls. **A**, **B** Individual data were shown. *P <  0.05 for analysis using Wilcoxon signed rank sum test

As shown, serum levels of IL-1β, IL-6, IL-8, and TNF-α concomitantly changed with disease state (Fig. 2A) compared to CRP and ANCA titers of the patients (Fig. 2B). Serum cytokine measurement revealed that changes of monocyte-derived proinflammatory cytokines such as IL-1β, IL-6, IL-8, and TNF-α were associated with disease state.

### Correlation analysis among immune cell subsets, age, BVAS, and serum cytokines

Additionally, we analyzed the correlation for the number of representative immune cell subsets with age, BVAS, and serum cytokines. The age of patients and HCs was not matched in this study. To determine the effect of age, we examined the correlation between age and the number of representative immune cell subsets in AAV patients and HCs (Supplementary Fig. 4). No significant correlation was observed between subjects’ age and the number of cell subsets, such as monocytes (Supplementary Fig. 4A), CD14^++^ CD16^+^ intermediate monocytes (Supplementary Fig. 4B), CD19^+^ B cells (Supplementary Fig. 4C), and plasma cells (Supplementary Fig. 4D). Though the number of plasma cells seems to be increased in patients over 80 years, a previous report has shown that plasma cell counts are inversely correlated with age [34]. Thus, age did not affect immuno-phenotyping data in this study. Then, we showed the correlation for baseline BVAS with monocytes (Supplementary Fig. 5A), CD14^++^ CD16^+^ intermediate monocytes (Supplementary Fig. 5B), CD19^+^ B cells (Supplementary Fig. 5C), and plasma cells (Supplementary Fig. 5D). The numbers of CD14^++^ CD16^+^ intermediate monocytes and CD19^+^ B cells were correlated with chest and nervous system components of BVAS, respectively. Correlations for cytokine levels with monocytes (Supplementary Fig. 6A), CD14^++^ CD16^+^ intermediate monocytes (Supplementary Fig. 6B), CD19^+^ B cells (Supplementary Fig. 6C), and plasma cells (Supplementary Fig. 6D) were shown. Though the correlations for serum cytokine levels with these cell subsets were not statistically significant, they tend to be longitudinally associated with disease activity.

## Discussion

This study examined baseline and chronological changes in comprehensive immuno-phenotyping in patients with AAV. We revealed that CD14^++^ CD16^+^ intermediate monocytes and plasma cells were strongly correlated with disease relapse. Changes in CD14^++^ CD16^+^ intermediate monocytes and plasma cells were more sensitive markers of disease relapse than changes in MPO/PR3-ANCA titer. In particular, the number of CD14^++^ CD16^+^ intermediate monocytes at relapse was significantly greater than that in remission or in HCs. Taken together, our data suggest that the number of peripheral CD14^++^ CD16^+^ intermediate monocytes may be useful for evaluating disease activity in AAV.

Although B cell count and ANCA titer increase are shown to be associated with relapse [35, 36], these are not recommended for monitoring treatment [3]. Von Borstel et al. have reported CD27^+^ CD38^hi^ B cell frequencies during remission were associated with the risk for relapse in GPA patients, suggesting more specific cell subsets may potentially be markers associated with disease relapse [37]. We showed that changes in plasma cells were strongly correlated with the reactivation of CNS disease. This hypothesis is supported by our recent finding of a significant correlation between circulating B cells and the CNS component of BVAS [21, 38]. CNS lesion may be a good indicator of the need for B cell depletion therapy in AAV.

Here, we showed that changes in CD14^++^ CD16^+^ intermediate monocytes were remarkably associated with disease relapse in AAV. Although CD14^++^ CD16^+^ intermediate monocytes account for a relatively small fraction of total monocytes in peripheral blood (about 8% of monocytes in HC) [21], the proportion of CD14^++^ CD16^+^ intermediate monocytes is increased to 15% among total monocytes in AAV patients [21, 39]. We validated the former analyses [21, 39] and identified CD14^++^ CD16^+^ intermediate monocyte as the most useful marker of disease relapse in AAV using comprehensive immuno-phenotyping. Though CD14^++^ CD16^+^ intermediate monocytes expansion have been reported in other inflammatory diseases such as rheumatoid arthritis [40], van Sleen et al. reported that CD14^++^ CD16^−^ classical monocytes expanded in GCA rather than CD14^++^ CD16^+^ intermediate monocytes [41]. Our recent report revealed that LVV disease activity was not consistent with changes of CD14^++^ CD16^+^ intermediate monocytes [42]. In combination with findings that monocytes and macrophages are frequently found in vascular infiltrates of AAV patients [43], CD14^++^ CD16^+^ intermediate monocytes may play a characteristically important role in the pathogenesis of AAV. Interestingly, the number of CD14^++^ CD16^+^ intermediate monocytes remained elevated in AAV patients who had achieved remission (Fig. 1B and Supplementary Fig. 2C). Considering the elevated level of CD14^++^ CD16^+^ intermediate monocytes was independent of the treatment regimen, the existing treatments might not be sufficient to achieve deep and stable remission state in AAV. Our findings raise the possibilities that pathogenic CD14^++^ CD16^+^ intermediate monocytes are resistant to the current treatments and controlling the functions of this cell subset may lead to reduction of relapse in AAV. Our study suggests that CD14^++^ CD16^+^ intermediate monocytes may be one of the novel therapeutic cell targets against AAV.

While the upstream of CD14^++^ CD16^+^ intermediate monocytes expansion or their molecular effect in AAV pathogenesis is beyond the scope of this manuscript, CD16, a receptor for immunoglobin gamma Fc region III, may have a key role in the pathogenesis of AAV. The proportion of CD16 on intermediate monocytes correlates with MPO expression on these cells [44], providing an insight into the possible activation of these cells by MPO-ANCA. ANCA has been shown to stimulate oxygen radical production and to produce inflammatory cytokines from monocytes [45, 46]. We found that the levels of IL-1β, IL-6, IL-8, and TNF-α were associated with disease activity. CD14^++^ CD16^+^ intermediate monocyte is the most proinflammatory monocyte subset and also involved in cell differentiation, including development from naive lymphocyte into Th17 [47]. Considering major sources of these cytokines are activated monocytes and macrophages, CD14^++^ CD16^+^ intermediate monocytes are key effectors in AAV pathogenesis through the production of proinflammatory cytokines.

Our study had several limitations. First, this is a study with a small sample size and with a short observation period. Second, treatment was not randomized and depends on the attending physician. Third, our cohort included relatively less kidney recurrence, whereas previous trials such as RITUXVAS and RAVE mainly focused on renal vasculitis [4, 5]. Last, we did not show that the changes in CD14^++^ CD16^+^ intermediate monocytes were specific for AAV because we did not have disease controls. For these reasons, this report represented a pilot study that was designed for discovery analysis and which had inadequate power to incorporate an adjustment for multivariate analysis. Further studies of additional immuno-phenotyping evidence are required to strengthen our findings and make it possible to predict disease relapse in AAV.

## Conclusions

Our findings demonstrate CD14^++^ CD16^+^ intermediate monocyte counts reflect the disease activity of AAV and will aid to distinguish active patients from those in remission. Chronological changes in CD14^++^ CD16^+^ intermediate monocyte counts can be a marker of disease relapse in AAV patients.

## Supplementary information

**Additional file 1: Supplementary Fig. 1**. Immuno-phenotyping strategy using antibody staining. Details of the gating strategies of (A) Panel 1 (helper T cell and follicular helper T cell), (B) Panel 2 (regulatory T cell), (C) Panel 3 (B cell) and (D) Panel 4 (monocyte, neutrophil and eosinophil).

**Additional file 2: Supplementary Fig. 2**. Chronological changes in immuno-phenotyping in AAV patients without relapse. Changes in (A) PSL dose, (B) BVAS, (C) CD14^++^ CD16^+^ intermediate monocytes and (D) plasma cells in AAV patients without relapse (n = 20). Lines showed the median (IQR) number of immune cell subsets in healthy controls.

**Additional file 3: Supplementary Fig. 3**. Changes in humoral factors according to treatment, ANCA serotype or disease subtype. Serum levels of (A) IFN-γ, (B) IL-1β, (C) IL-6, (D) IL-8, (E) IL-10, (F) TNF-α, (G) CRP and (H) ANCA titer in patients with relapse (n = 9). (A-F) Lines showed the median (IQR) level of cytokines in healthy controls. Difference among treatment regimens (A-b, B-b, C-b, D-b, E-b, F-b, G-b and H-b) and disease phenotypes (A-c, B-c, C-c, D-c, E-c, F-c, G-c and H-c) were analyzed by repeated measures ANOVA and post-hoc Friedman test.

**Additional file 4: Supplementary Fig. 4**. Correlation between age and number of representative immune cell subsets. Correlation between age and the number of (A) monocytes, (B) CD14^++^ CD16^+^ intermediate monocytes, (C) CD19^+^ B cells and (D) plasma cells. (A-a, B-a, C-a, D-a) HC; (A-b, B-b, C-b, D-b) AAV. Pearson’s correlation coefficient was used.

**Additional file 5: Supplementary Fig. 5**. Correlation for baseline BVAS with representative immune cell subsets. Correlation for baseline BVAS with (A) monocytes, (B) CD14^++^ CD16^+^ intermediate monocytes, (C) CD19^+^ B cells and (D) plasma cells. (a) Total BVAS, and components of BVAS for (b) ear, nose, throat, (c) nervous system, (d) renal and (e) chest were shown. Pearson’s correlation coefficient was used.

**Additional file 6: Supplementary Fig. 6**. Correlations for cytokine levels with representative immune cell subsets. Correlations for cytokine levels with (A) monocytes, (B) CD14^++^ CD16^+^ intermediate monocytes, (C) CD19^+^ B cells and (D) plasma cells. Correlations for each cytokine of (a) IFN-γ, (b) IL-1β, (c) IL-6, (d) IL-8, (e) IL-10 and (f) TNF-α are shown. Pearson’s correlation coefficient was used.

**Additional file 7:** Supplementary Table 1. Antibodies used in FACS analysis.

## Data Availability

All data generated and analyzed in this study are disclosed.

## Abbreviations

ANCA: Anti-neutrophil cytoplasmic antibody
AAV: ANCA-associated vasculitis
csDMARDs: Conventional disease-modifying anti-rheumatic drugs
HLA: Human leukocyte antigen
MHC: Major histocompatibility
MPO: Myeloperoxidase
Th: Helper T
Tfh: Follicular helper T
GPA: Granulomatosis with polyangiitis
MPA: Microscopic polyangiitis
PSL: Prednisolone
ENT: Ear, nose and throat
CNS: Central nervous system
PNS: Peripheral nervous system
ILD: Interstitial lung disease
CLEIA: Chemiluminescent enzyme immunoassay
RF: Rheumatoid factor
BVAS: Birmingham Vasculitis Activity Score
IFN: Interferon
IL: Interleukin
TNF: Tumor necrosis factor
LVV: Large vessel vasculitis
