# Supplementary figures and images for: Longitudinal immune cell monitoring identified CD14++ CD16+ intermediate monocyte as a marker of relapse in patients with ANCA-associated vasculitis
Source: Arthritis Res Ther. 2020 Jun 16;22:145. doi: 10.1186/s13075-020-02234-8 (PMC7298936; doi:10.1186/s13075-020-02234-8)

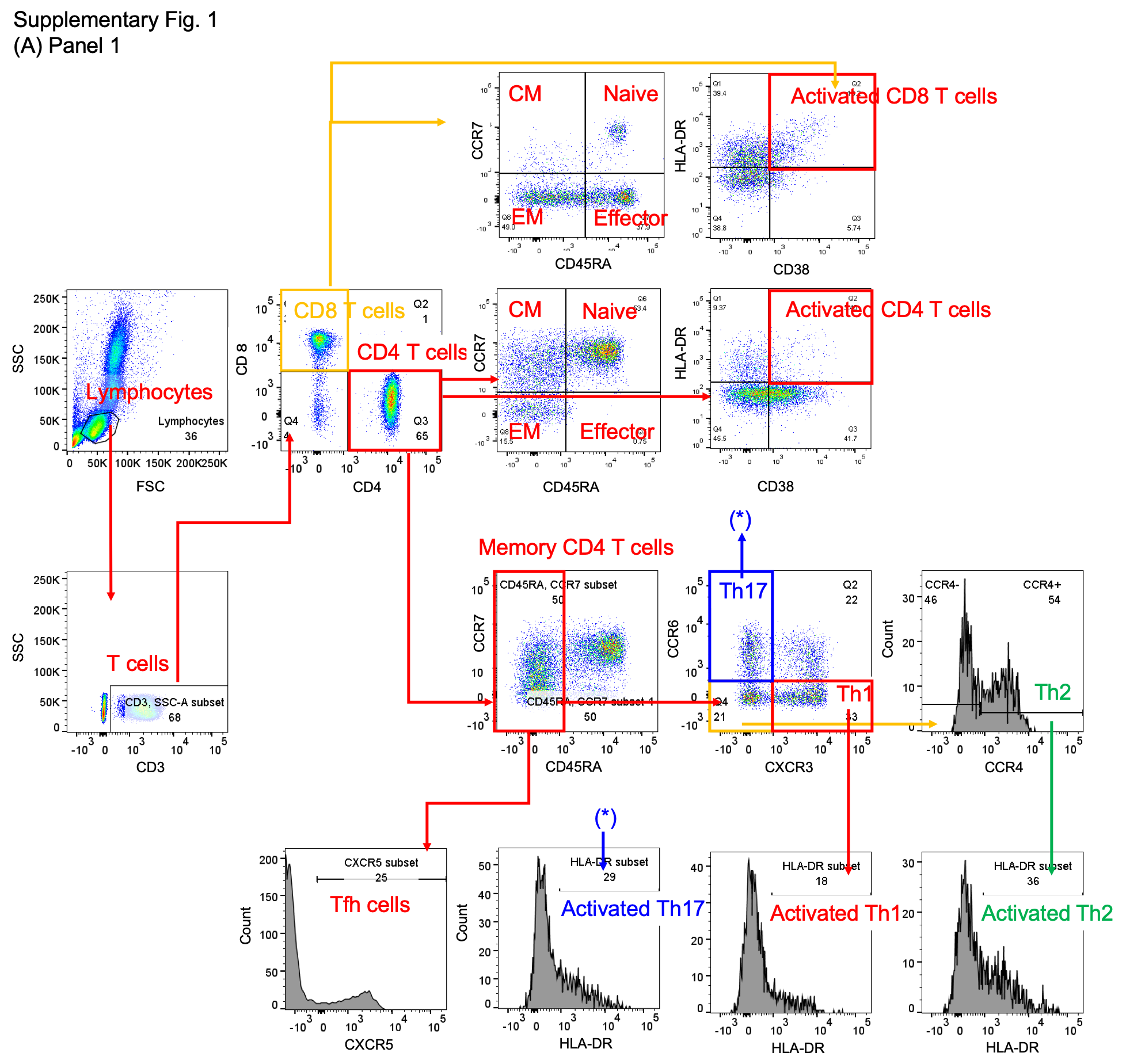

Supplement: Supplementary file 1 — Additional file 1: Supplementary Fig. 1. Immuno-phenotyping strategy using antibody staining. Details of the gating strategies of (A) Panel 1 (helper T cell and follicular helper T cell), (B) Panel 2 (regulatory T cell), (C) Panel 3 (B cell) and (D) Panel 4 (monocyte, neutrophil and eosinophil). [file 13075_2020_2234_MOESM1_ESM.zip › Supplementary Fig. 1A.png]

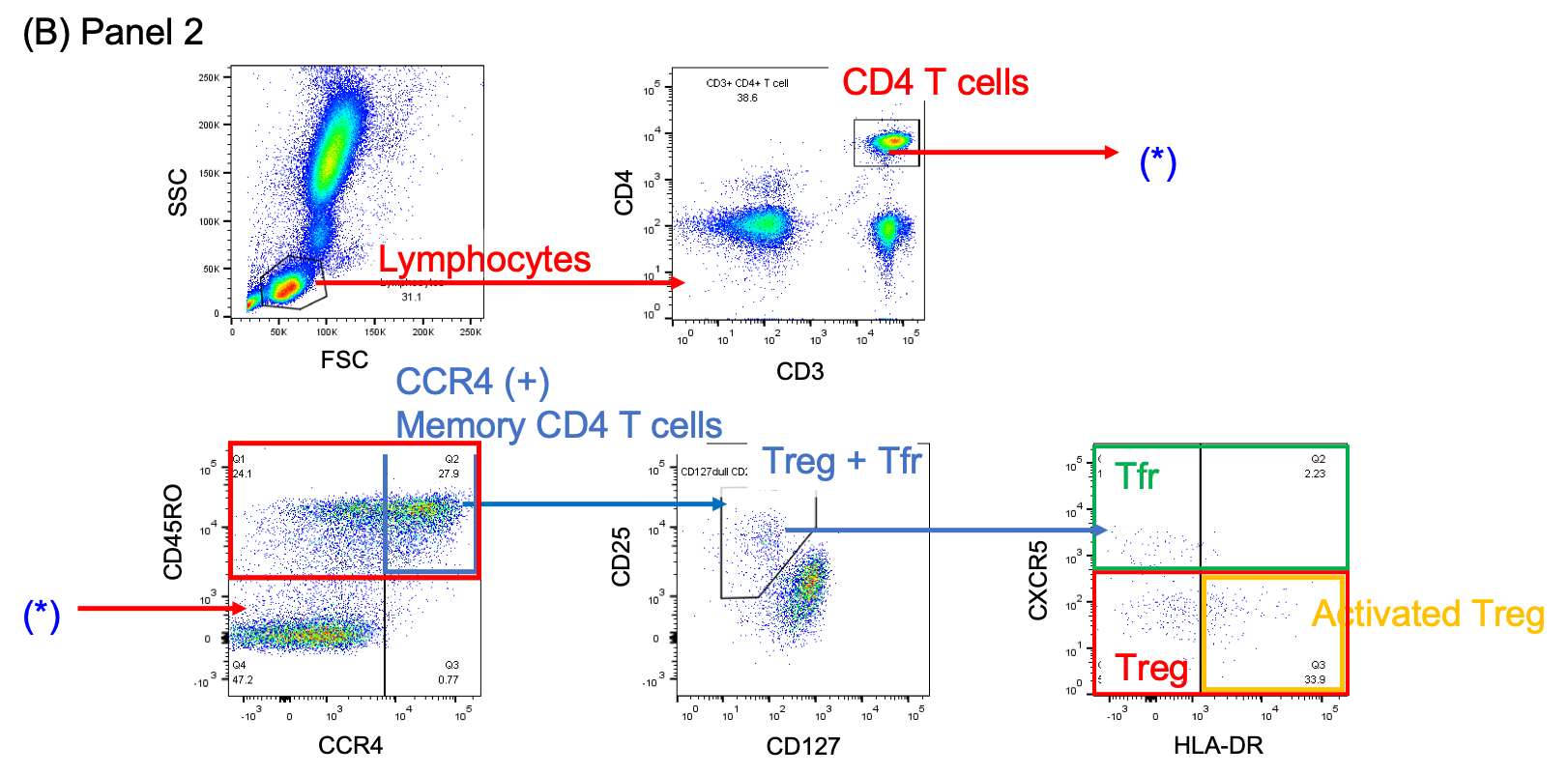

Supplement: Supplementary file 1 — Additional file 1: Supplementary Fig. 1. Immuno-phenotyping strategy using antibody staining. Details of the gating strategies of (A) Panel 1 (helper T cell and follicular helper T cell), (B) Panel 2 (regulatory T cell), (C) Panel 3 (B cell) and (D) Panel 4 (monocyte, neutrophil and eosinophil). [file 13075_2020_2234_MOESM1_ESM.zip › Supplementary Fig. 1B.png]

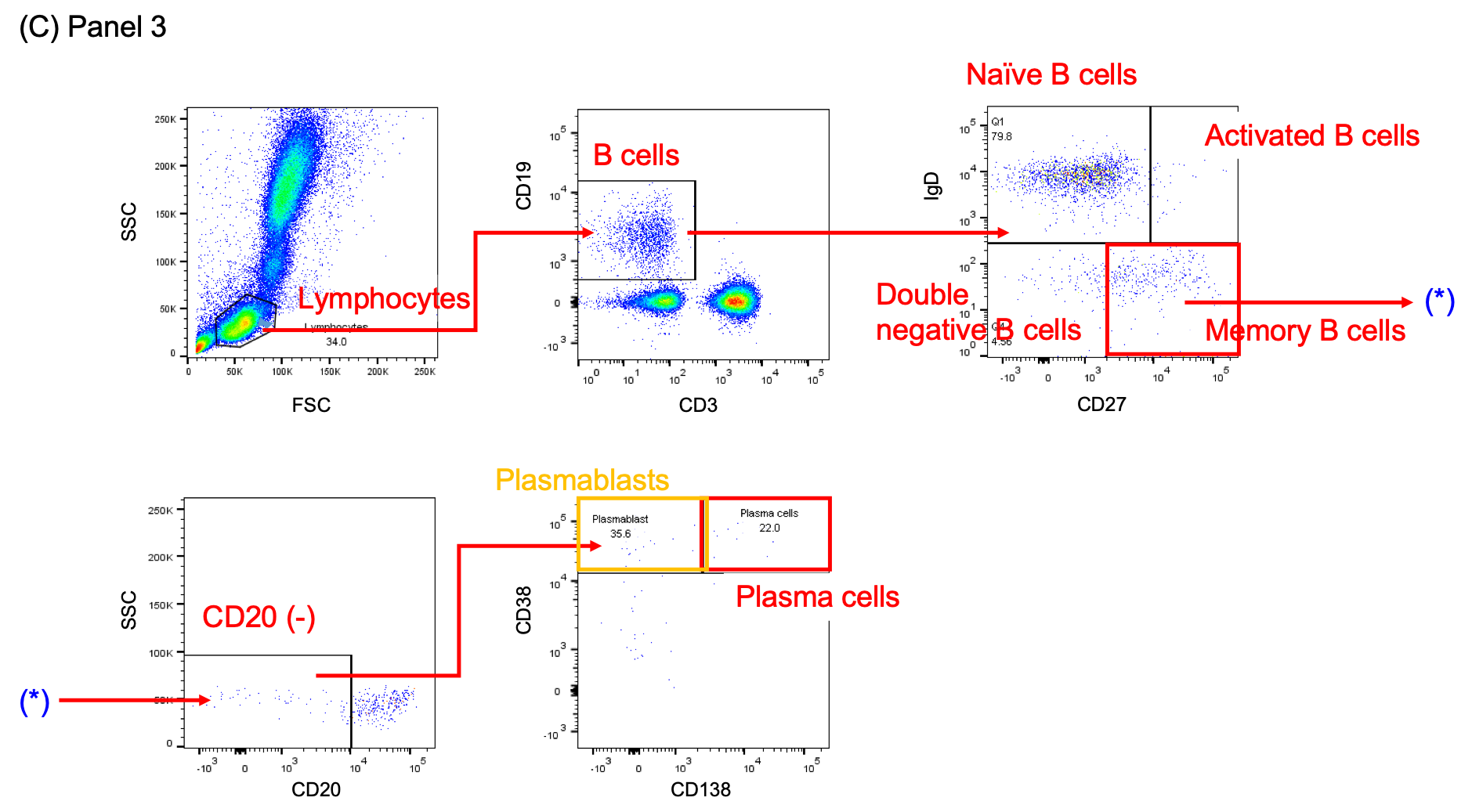

Supplement: Supplementary file 1 — Additional file 1: Supplementary Fig. 1. Immuno-phenotyping strategy using antibody staining. Details of the gating strategies of (A) Panel 1 (helper T cell and follicular helper T cell), (B) Panel 2 (regulatory T cell), (C) Panel 3 (B cell) and (D) Panel 4 (monocyte, neutrophil and eosinophil). [file 13075_2020_2234_MOESM1_ESM.zip › Supplementary Fig. 1C.png]

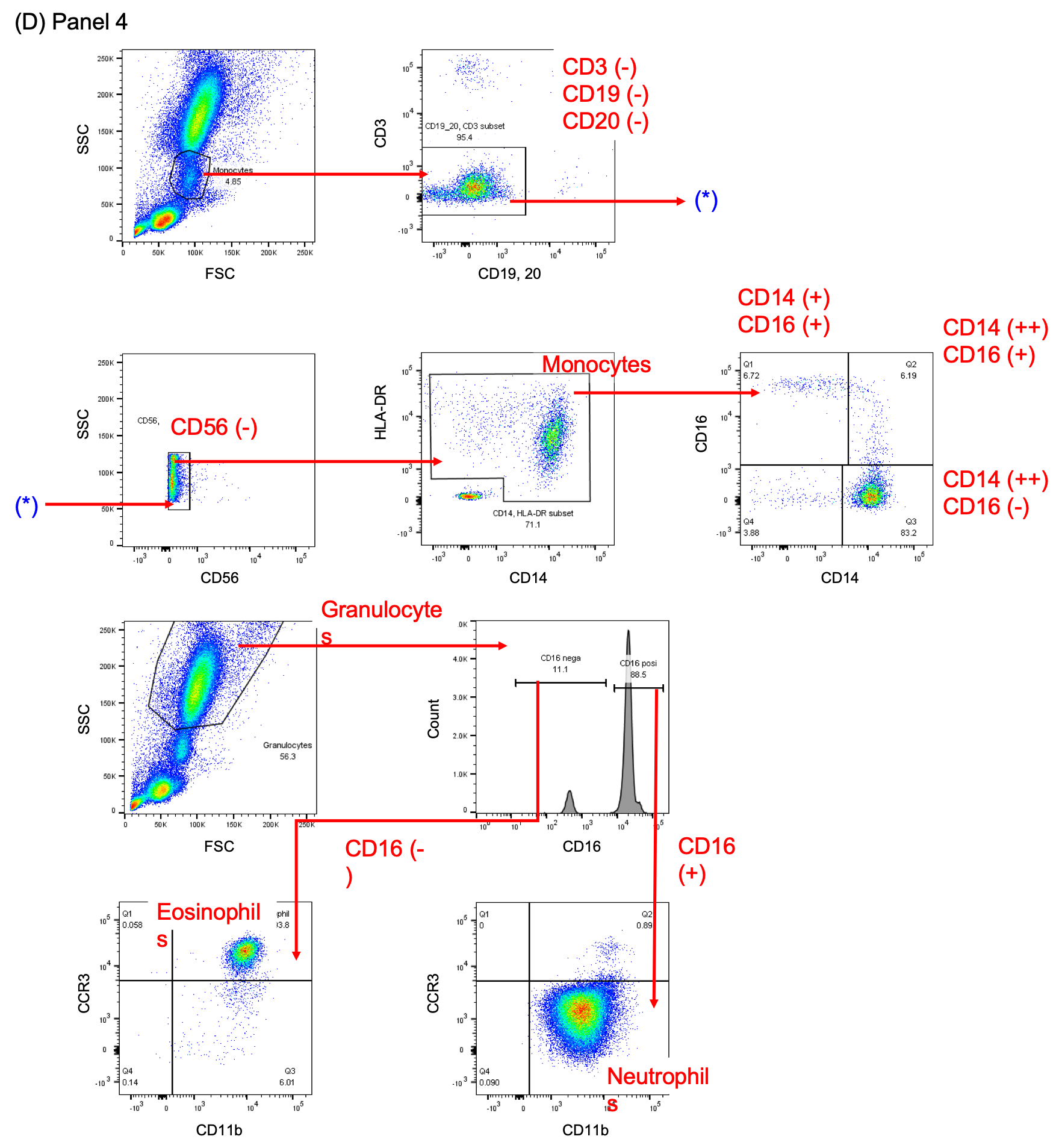

Supplement: Supplementary file 1 — Additional file 1: Supplementary Fig. 1. Immuno-phenotyping strategy using antibody staining. Details of the gating strategies of (A) Panel 1 (helper T cell and follicular helper T cell), (B) Panel 2 (regulatory T cell), (C) Panel 3 (B cell) and (D) Panel 4 (monocyte, neutrophil and eosinophil). [file 13075_2020_2234_MOESM1_ESM.zip › Supplementary Fig. 1D.png]

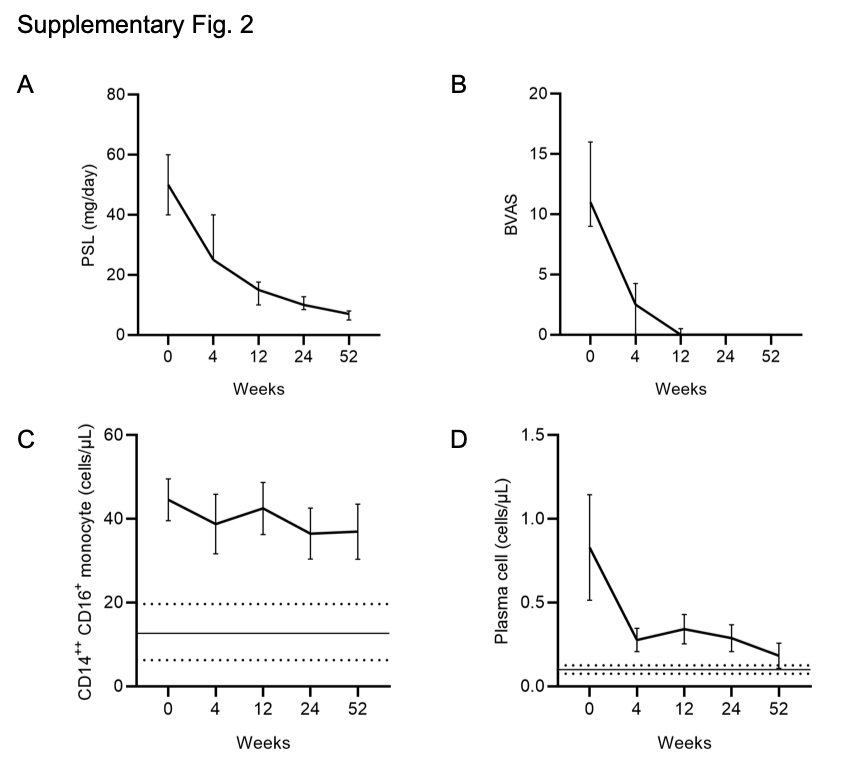

Supplement: Supplementary file 2 — Additional file 2: Supplementary Fig. 2. Chronological changes in immuno-phenotyping in AAV patients without relapse. Changes in (A) PSL dose, (B) BVAS, (C) CD14++ CD16+ intermediate monocytes and (D) plasma cells in AAV patients without relapse (n = 20). Lines showed the median (IQR) number of immune cell subsets in healthy controls. [file 13075_2020_2234_MOESM2_ESM.png]

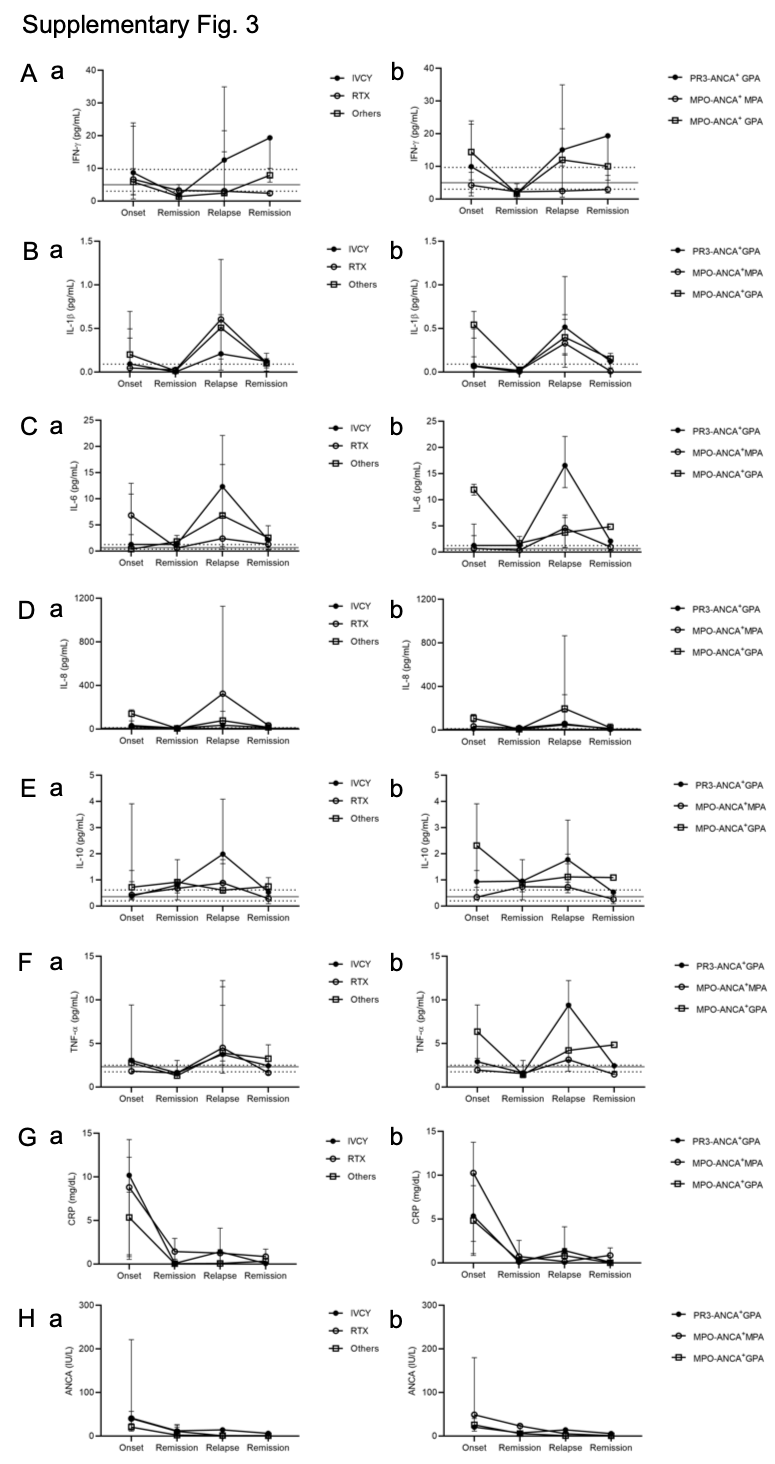

Supplement: Supplementary file 3 — Additional file 3: Supplementary Fig. 3. Changes in humoral factors according to treatment, ANCA serotype or disease subtype. Serum levels of (A) IFN-γ, (B) IL-1β, (C) IL-6, (D) IL-8, (E) IL-10, (F) TNF-α, (G) CRP and (H) ANCA titer in patients with relapse (n = 9). (A-F) Lines showed the median (IQR) level of cytokines in healthy controls. Difference among treatment regimens (A-b, B-b, C-b, D-b, E-b, F-b, G-b and H-b) and disease phenotypes (A-c, B-c, C-c, D-c, E-c, F-c, G-c and H-c) were analyzed by repeated measures ANOVA and post-hoc Friedman test. [file 13075_2020_2234_MOESM3_ESM.png]

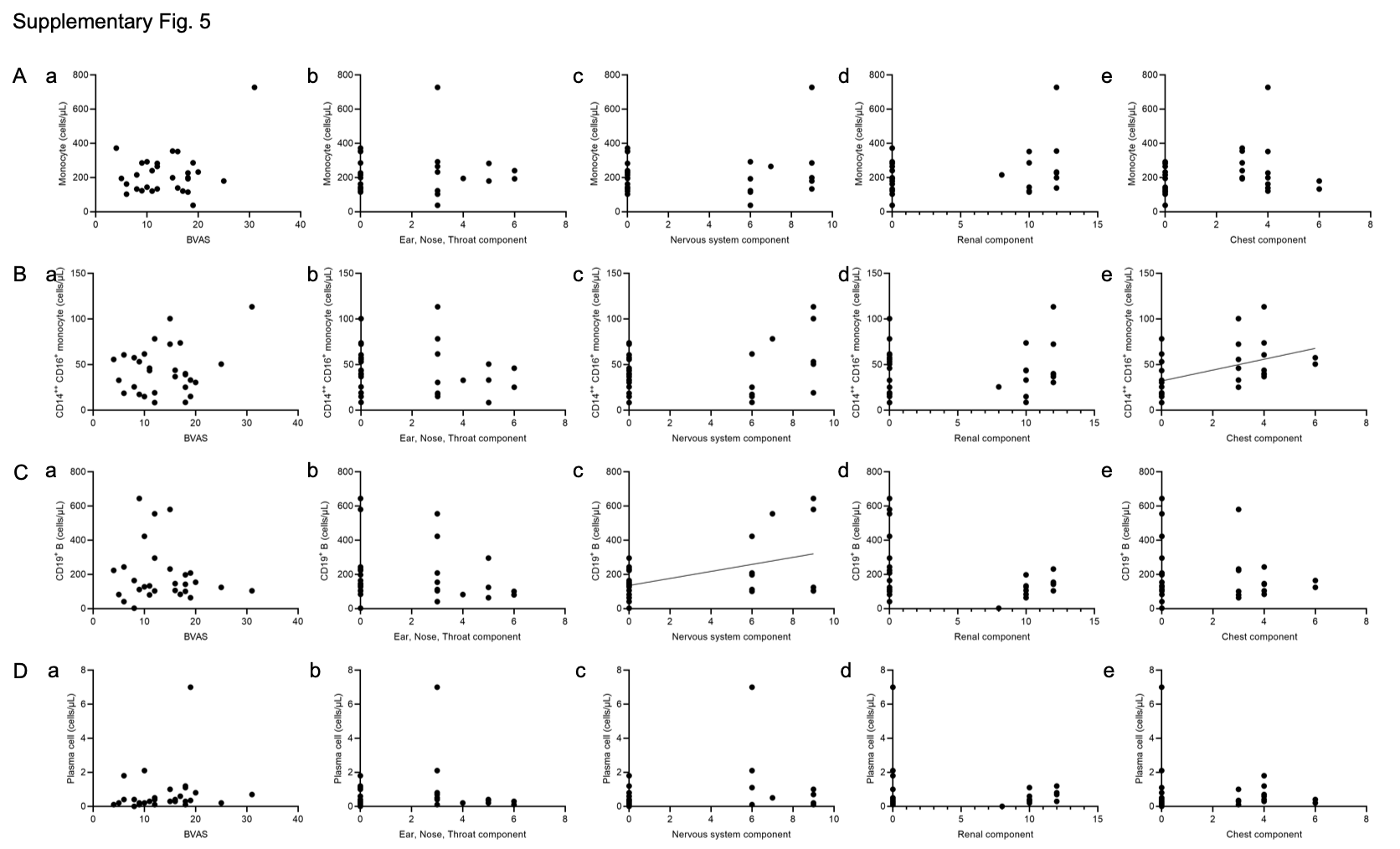

Supplement: Supplementary file 5 — Additional file 5: Supplementary Fig. 5. Correlation for baseline BVAS with representative immune cell subsets. Correlation for baseline BVAS with (A) monocytes, (B) CD14++ CD16+ intermediate monocytes, (C) CD19+ B cells and (D) plasma cells. (a) Total BVAS, and components of BVAS for (b) ear, nose, throat, (c) nervous system, (d) renal and (e) chest were shown. Pearson’s correlation coefficient was used. [file 13075_2020_2234_MOESM5_ESM.png]

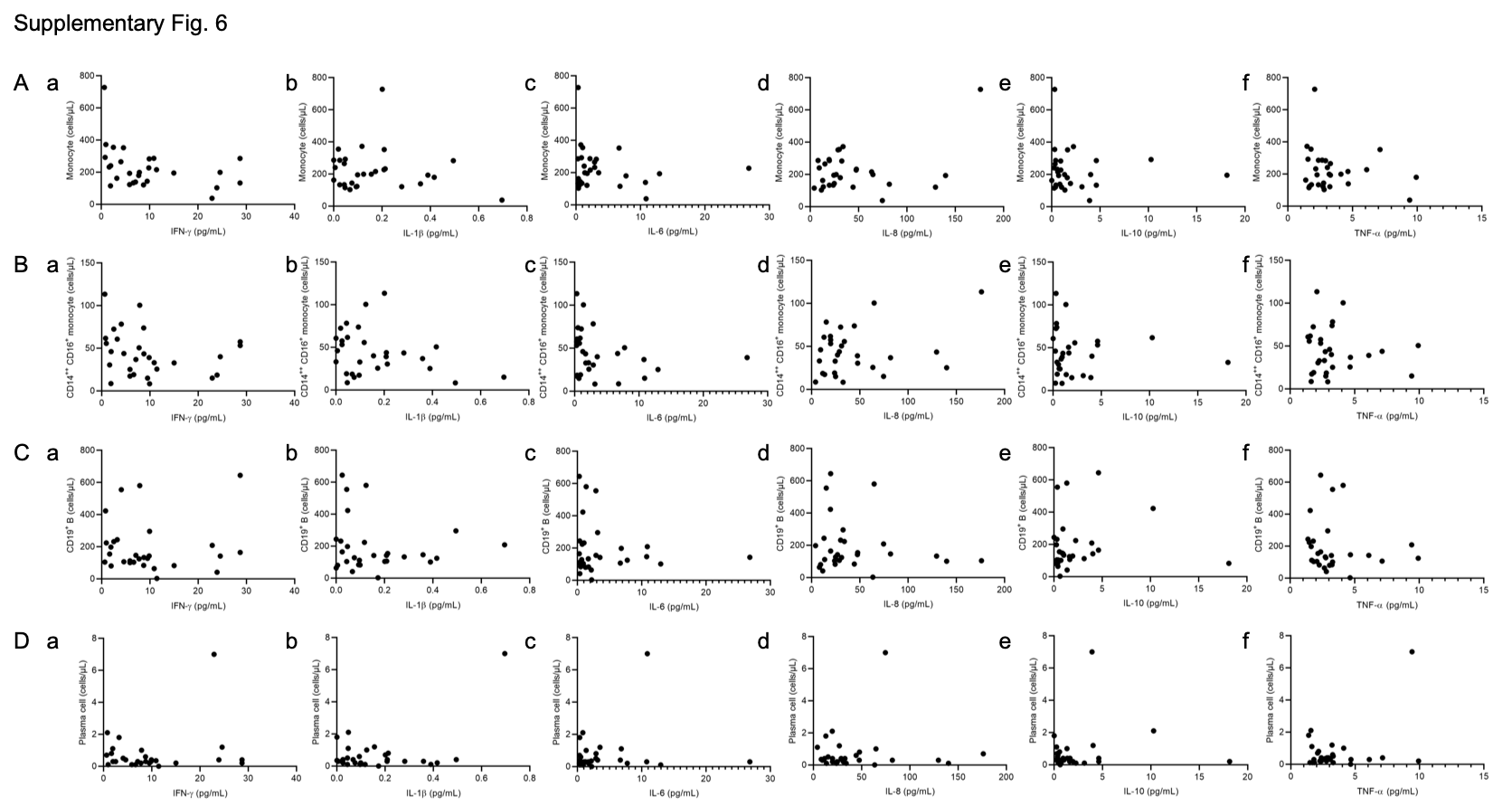

Supplement: Supplementary file 6 — Additional file 6: Supplementary Fig. 6. Correlations for cytokine levels with representative immune cell subsets. Correlations for cytokine levels with (A) monocytes, (B) CD14++ CD16+ intermediate monocytes, (C) CD19+ B cells and (D) plasma cells. Correlations for each cytokine of (a) IFN-γ, (b) IL-1β, (c) IL-6, (d) IL-8, (e) IL-10 and (f) TNF-α are shown. Pearson’s correlation coefficient was used. [file 13075_2020_2234_MOESM6_ESM.png]
