# Supplementary material for: Longitudinal immune cell monitoring identified CD14++ CD16+ intermediate monocyte as a marker of relapse in patients with ANCA-associated vasculitis
Source: Arthritis Res Ther. 2020 Jun 16;22:145. doi: 10.1186/s13075-020-02234-8 (PMC7298936; doi:10.1186/s13075-020-02234-8)
Supplement: Supplementary file 7 — Additional file 7: Supplementary Table 1. Antibodies used in FACS analysis. [file 13075_2020_2234_MOESM7_ESM.docx]

Supplementary Table 1. Antibodies used in FACS analysis.

| Fluorecent dye | Panel 1 (helper T cell and follicular helper T cell) | | Panel 2 (regulatory T cell) | |
| --- | --- | --- | --- | --- |
|  | Anti-human surface antigen antibody | Clone | Anti-human surface antigen antibody | Clone |
| FITC | CD3 | UCHT1 | CD127 | HIL-7R-M21 |
| PE | CXCR3 | 1C6/CXCR3 | CD25 | M-A251 |
| PE-CF594 | CCR7 | 150503 | CD3 | UCHT1 |
| PC5.5 | HLA-DR | G46-6 | HLA-DR | G46-6 |
| PC7 | CD45RA | HI100 | CCR4 | 1G1 |
| APC | CD38 | HIT2 |  |  |
| APC-Cy7 | CD8 | RPA-T8 | CD45RO | UCHL1 |
| BV421 | CXCR5 | RF8B2 | CXCR5 | RF8B2 |
| BV510 |  |  |  |  |
| BV605 | CCR4 | 1G1 |  |  |
| BV711 | CCR6 | G034E3 | CD8 | RPA-T8 |
| BV786 | CD4 | SK3 | CD4 | SK3 |
| Fluorecent dye | Panel 3 (B cell) | | Panel 4 (monocyte, neutrophil and eosinophil) | |
|  | Anti-human surface antigen antibody | Clone | Anti-human surface antigen antibody | Clone |
| FITC | CD3 | UCHT1 | CD56 | B156 |
| PE |  |  | NKG2D | 1D11 |
| PE-CF594 | HLA-DR | G46-6 | CCR3 | 5E8 |
| PC5.5 | CD19 | HIB19 | CD123 | 7G3 |
| PC7 | CD27 | M-T271 | CD11c | B-ly6 |
| APC | CD38 | HIT2 | CD16 | 3G8 |
| APC-Cy7 | CD20 | 2H7 | CD19/CD20 | HIB19/2H7 |
| BV421 |  |  |  |  |
| BV510 | IgD | IA6-2 | HLA-DR | L243 |
| BV605 | CD138 | MI15 | CD14 |  |
| BV711 |  |  | CD11b | ICRF44 |
| BV786 |  |  | CD3 | SK7 |
