# Supplementary material for: Longitudinal immune cell monitoring identified CD14++ CD16+ intermediate monocyte as a marker of relapse in patients with ANCA-associated vasculitis
Source: Arthritis Res Ther. 2020 Jun 16;22:145. doi: 10.1186/s13075-020-02234-8 (PMC7298936; doi:10.1186/s13075-020-02234-8)

Supplementary Fig. 4

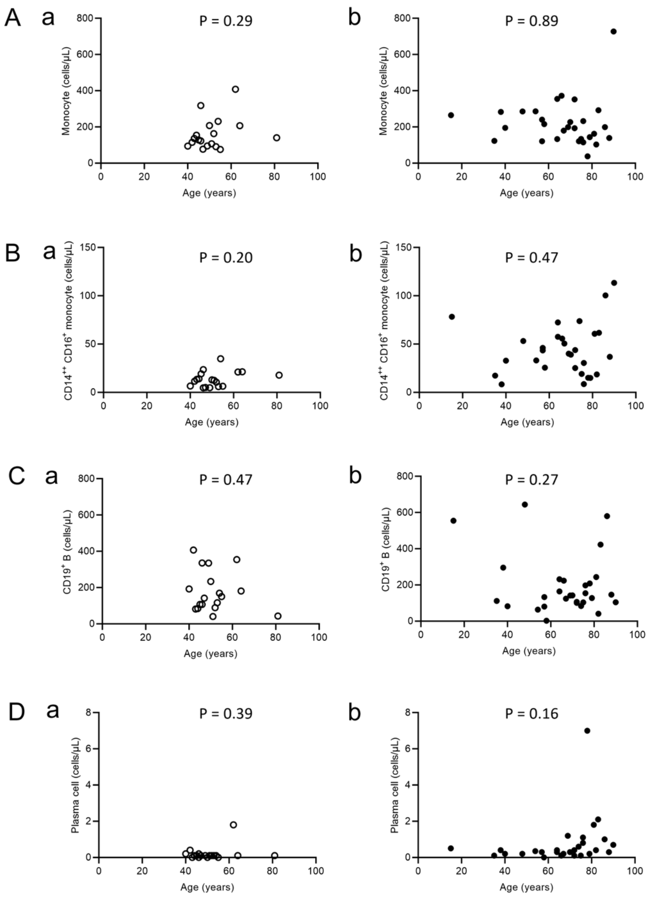

Supplement: Supplementary file 4 [file 13075_2020_2234_MOESM4_ESM.docx]
